# Nano-precision metrology of X-ray mirrors with laser speckle angular measurement

**DOI:** 10.1038/s41377-021-00632-4

**Published:** 2021-09-22

**Authors:** Hongchang Wang, Simone Moriconi, Kawal Sawhney

**Affiliations:** 1 Diamond Light Source Ltd, Harwell Science and Innovation Campus, Didcot, OX11 0DE UK; 2 Department of Engineering Science, University of Oxford, Parks Road, Oxford, OX1 3PJ UK

**Keywords:** Optical sensors, Photonic devices, Diode lasers

## Abstract

X-ray mirrors are widely used for synchrotron radiation, free-electron lasers, and astronomical telescopes. The short wavelength and grazing incidence impose strict limits on the permissible slope error. Advanced polishing techniques have already produced mirrors with slope errors below 50 nrad root mean square (rms), but existing metrology techniques struggle to measure them. Here, we describe a laser speckle angular measurement (SAM) approach to overcome such limitations. We also demonstrate that the angular precision of slope error measurements can be pushed down to 20nrad rms by utilizing an advanced sub-pixel tracking algorithm. Furthermore, SAM allows the measurement of mirrors in two dimensions with radii of curvature as low as a few hundred millimeters. Importantly, the instrument based on SAM is compact, low-cost, and easy to integrate with most other existing X-ray mirror metrology instruments, such as the long trace profiler (LTP) and nanometer optical metrology (NOM). The proposed nanometrology method represents an important milestone and potentially opens up new possibilities to develop next-generation super-polished X-ray mirrors, which will advance the development of X-ray nanoprobes, coherence preservation, and astronomical physics.

## Abstract (web summary)

A versatile high precision metrology instrument has been developed that surpass the limits of existing metrology techniques and opens up new possibilities to develop next-generation super-polished X-ray mirrors.

## Graphical abstract

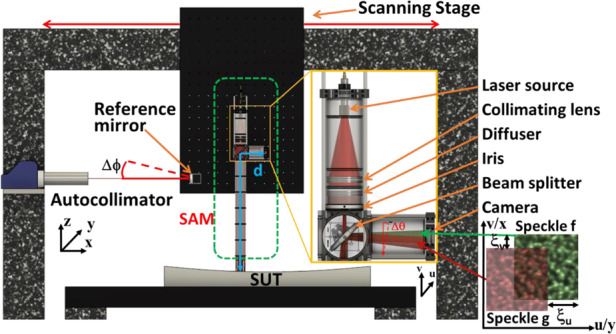

## Introduction

Modern synchrotron radiation facilities and X-ray free-electron lasers provide high-brilliance X-rays for cutting-edge scientific and industrial research, which explores the world through a refined understanding of the structure of matter. X-ray telescopes allow astronomers to measure the universe’s faint X-ray sources and the distribution of heat in celestial bodies between galaxies^1^. The successful exploitation and efficient utilization of X-ray beams depend on the quality of the optics. Among these X-ray optics, X-ray mirrors are critical optical components and are widely used for their exceptional characteristics of high efficiency and inherent achromaticity^2^. According to Snell’s Laws or the Bragg Equation, X-ray mirrors must be operated at grazing incidence by either total external reflection from a single-layer coating or Bragg reflection from a multilayer coating^3^. The height error (surface deviations from the ideal profile) of X-ray mirrors inevitably deteriorates the wavefront and focal performance. The extremely stringent tolerances on height error for X-ray mirrors are a consequence of Rayleigh’s quarter-wavelength rule applied to X-ray wavelengths less than one-thousandth of those for visible light^2^. For the most demanding X-ray applications such as extreme energy resolution or nanofocusing, the required height error is often below 1 nm rms^3–5^. The manufacturing and metrology of X-ray mirrors thus poses major challenges.

Over the last few decades, continuous progress has been made in improving both the accuracy and precision of metrology and manufacturing techniques to improve the optical quality of X-ray mirrors. Particularly noteworthy has been the development of deterministic surface finishing techniques such as elastic emission machining (EEM)^6^ and ion beam figuring^7^, which have enabled the fabrication of mirror surfaces with sub-nm height errors and slope errors (slope deviations from the ideal shape) below 50 nrad rms. However, even these high-precision optics are not enough to keep pace with the worldwide upgrades of synchrotrons to diffraction-limited storage rings^6^, or with the excellent spatial and temporal coherence of X-ray free-electron lasers, or with the increasing demand for high-quality aspheric focusing mirrors with radii of curvature down to a few hundred millimetres for next-generation X-ray telescopes^7–10^. Currently available metrology techniques are not adequate to guide the latest efforts to improve the manufacturing quality of X-ray mirrors, and at the same time, the transfer of the metrology data to the manufacturing process is also presenting challenges. More accurate measurements of mirror figures are indispensable if next-generation X-ray mirrors capable of taking advantage of these improved sources and meeting the new demands are to be successfully made.

Current common metrology methods for X-ray mirror figures can be divided into those based on stitching and those based on “pencil beam” (deflectometry). These methods differ vastly in precision, measurement speed, angle range, experimental setup, and signal recorded. Stitching methods, such as relative angle determinable stitching interferometry (RADSI)^8^, the stitching Shack Hartmann optical Head (SSH-OH)^9^, or tilt-measurement-based stitching interferometry (TSI)^10^, depend on the measurement of multiple small overlapping regions to overcome the limited instrumental range of a single two-dimensional (2D) figure measurement. However, the process of stitching adjacent regions together is often complicated and is affected by cumulative systematic errors. Pencil-beam devices, such as the long trace profiler (LTP), nanometer optical metrology (NOM), and their successive versions^11–17^, are widely used, simple to interpret, and able to measure X-ray mirrors up to 1.5 m long, but only along one dimension (1D) at a time. Each successive version has brought improved precision to the figure measurements, so that today’s NOM can inspect the figure of flat or weakly curved mirrors with remarkable precision: down to 50 nrad^18–20^. Nonetheless, despite the use of a penta-prism to keep the beam deflection independent of the slide’s pitch error, the sawtooth deviation (over 250 nrad rms) from the electronic autocollimator (AC) interferes with the NOM’s measurement of the angle of deflection of the surface under test, and special calibration should be used to minimize the sawtooth error^21^. In addition, the NOM’s limited slope measurement range keeps it from measuring surfaces with radii of curvature less than 5 m^12^.

To overcome the above limitations of present metrology techniques, we propose a novel metrology instrument, the speckle angular measurements (SAM) optical scanning head. 2D random intensity patterns (speckle) are generated by shining a laser through a diffuser and they can be treated as multiple pencil beams with different features. Variations of mirror slope over the measured area of the mirror shift the speckle pattern. The slope variation of the surface under test (SUT) can then be measured at the nanoradian level in two dimensions by precisely tracking the speckle displacement with an advanced sub-pixel algorithm. The instrument based on SAM is compact, low-cost, and easy to integrate with most existing pencil beam-based metrology instruments, such as the LTP and the NOM. Moreover, while the autocollimator in the NOM measures only a few peaks from the multislit reticle, the SAM measures a very large number of speckles, thus providing much better statistics and less random noise even in a single image. All these remarkable features will potentially enable the proposed SAM metrology technique to be widely used for super-precision metrology and the advancement of the next generation of X-ray mirrors.

**Fig. 1:**
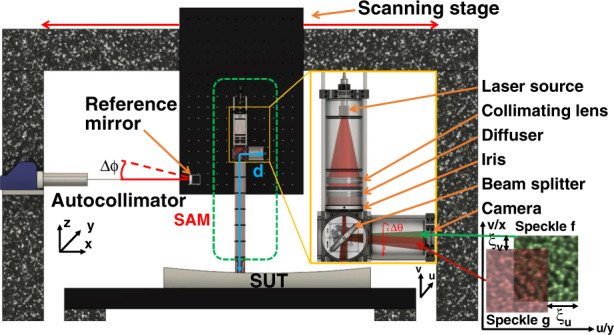
Schematic representation of the experimental setup for speckle angular measurement (SAM). The SAM instrument is attached to the air-bearing scan stage, and the speckle pattern from the reflected mirror surface shifts when the speckle is projected onto different parts of the surface under test (SUT). The angle change can be calculated from the speckle displacement between two speckle subset images (f and g) by employing the sub-pixel tracking algorithm. The carriage slide’s pitch error is measured with an autocollimator, and the change of the SUT’s slope is derived from the SAM and the autocollimator (AC) angle

Ever since the invention of laser light, speckle has been explored extensively and used for strain and vibration measurement, holographic interferometry, and speckle imaging^22^. Recently, X-ray near-field speckle has been successfully applied to in-situ and at-wavelength metrology of X-ray optics and advanced X-ray imaging^23–31^. However, although measurements with synchrotron X-rays are ideal, synchrotron beamlines are often oversubscribed, and applications for beamtime may take weeks or months^32^. Synchrotron beamlines are also not readily accessible to industrial manufacturers of optics. To provide an off-beamline facility, we have built a SAM metrology instrument with a visible light laser. The principle of SAM is presented in Fig. 1.

The SAM instrument is retrofitted on an existing NOM gantry (Supplementary Fig. S1). The SAM is composed of a laser, a collimating objective lens, a diffuser, an iris, a beam-splitter, and a high-resolution camera. To improve the stability and minimize the power dissipation, a low-power laser diode (4.5 mW at 635 nm) was used. The laser was firstly collimated with a collimating lens and then passed through a diffuser. Two-dimensional (2D) objective speckle patterns can be formed when the scattered laser light falls on the mirror surface. To achieve the best speckle tracking accuracy with the objective speckle pattern, the mean intensity gradient (MIG) is used to assess the quality of various diffusers^33^. A piece of invisible tape is finally selected as the diffuser because its speckle patterns have a larger MIG than those of other diffusers (Supplementary Fig. S2). Note that the light at a given point in the speckle pattern is made up of contributions from the whole of the diffuser surface. An iris is attached just below the diffuser and used to adjust the scattering beam size and improve the spatial resolution of the test mirror surface.

The laser beam passes through the beam-splitter (50:50) so that part of it projects onto the mirror surface. The beam path is sealed with a tube to minimize the air turbulence. The beam reflected by the surface under test (SUT), still carrying the speckle pattern, is recorded by the high-resolution camera. Here, a mini camera (power consumption under 1 W, MU9PC-MH, Ximea Ltd) with a small housing (15 mm × 15 mm × 8 mm) is used to make it easy to fit to the system and minimize the power dissipation. The camera pixel size is 2.2 μm and the sensor’s active area is 5.7 mm × 4.3 mm. The mean speckle size (ss) at the camera plane can be expressed as

1 $$ss = \frac{{\lambda L}}{D}$$

where *λ* is the wavelength of the light, *D* is the aperture of the iris, and *L* is the distance traversed by the laser beam from the iris to the camera via the SUT and the beam splitter. As expected, the mean speckle size decreases with an increasing aperture of the iris but increases with increasing distance between the SAM and the SUT.

As the SAM instrument is translated by using the scanning carriage, the beam is projected onto different regions of the SUT. Here, the speckle pattern acts as a carrier of the SUT surface information. If the surface is of high quality without any discontinuities in slope, the speckle pattern recorded by the camera undergoes only a shift dependent on the SUT’s slope variation. Because each speckle pattern has unique features, the speckle may be treated as a set of multiple wavefront markers. The carriage slide’s pitch error Δϕ adds a systematic error because it too shifts the speckle. To monitor and correct for this error, a reference visible-light mirror is attached to the scanning stage to back-reflect the light beam from the autocollimator (AC) fixed on one side of the carriage’s slide. With a high-precision air-bearing translation stage, the slide’s pitch error can be kept down to tens of micro-radians. In this way, the AC’s sawtooth error can be avoided. The precision can be pushed down to 10nrad by increasing the averaging time and number of averaging scans^10^.

The description of the speckle tracking uses the two coordinate systems shown in Fig. 1:

- (*x,y*) are, respectively, the coordinates along the length (tangential direction) and the width (sagittal direction) of the SUT. They are in units of length and may take real values.
- (*u,v*) are, respectively, the coordinates along the horizontal direction and the vertical direction of the camera. They are in units of pixels and may take integer values only.

Within a single speckle image, a displacement (*δu,δv*) between two pixels on the camera is mapped into a displacement between the corresponding observation points (*δx,δy*) on the SUT:

2 $$\left\{ {\begin{array}{*{20}{c}} {\delta x = \varGamma _x\delta v} \\ {\delta y = \varGamma _y\delta u} \end{array}} \right.$$

*Γ*_*x*_ and *Γ*_*y*_ are scaling factors that depend on the divergence of the beam from the diffuser. They can be determined experimentally, as will be demonstrated later.

**Fig. 2:**
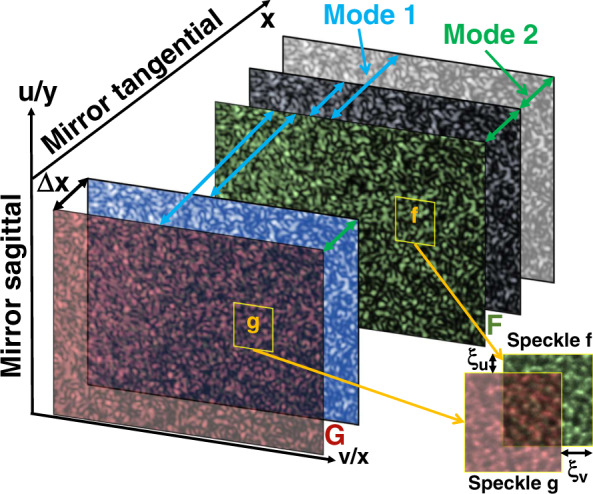
Illustration of 2 data processing modes and coordinate axes and main definitions. *F* and *G* are, respectively, the speckle image at the reference position and at the measured position on the SUT. *f* and *g* are corresponding subsets of *F* and *G*. *u* and *v* are, respectively, the horizontal and vertical coordinates of the camera. *x* and *y* are, respectively, the tangential and sagittal coordinates of the mirror’s SUT. ∆*x* is the scanning step size

Two data processing modes for linear scans of the SAM head along the SUT are proposed and described in Fig. 2. For Mode 1, a speckle image taken at a chosen region on the SUT is selected as the reference *F*(*u,v*), and the images *G*(*u,v*) taken on all the other regions of the SUT are compared with it. This yields a direct measurement of the first derivative of the surface height, which is the slope. For Mode 2, the speckle image *F*(*u,v*) recorded at any one point of the scan along the SUT is compared with the image *G*(*u,v*) recorded at the next point. The speckle displacement between these two consecutive images is related to the second derivative of the surface height, that is, the reciprocal of the local radius of curvature. In both modes, the speckle patterns in *F*(*u,v*) and *G*(*u,v*) are divided into the same grid of subsets as shown in Fig. 2 to provide the spatial resolution. A subset f centered at (*u*_*c*_*,v*_*c*_) with a size (2*M*_*f*_ + 1) × (2*N*_*f*_ + 1) is selected from (*u,v*), and a subset g with the same center (*u*_*c*_*,v*_*c*_) and with a size (2*M*_*g*_ + 1) × (2*N*_*g*_ + 1) is selected from *G*(*u,v*). To allow tracking, *M*_*g*_ > *M*_*f*_ and *N*_*g*_ > *N*_*f*_. In practice, *g* will have 10–20 more rows and columns than *f* for Mode 2. For Mode 1, the speckle displacements will be greater than in Mode 2 and so *g* must be correspondingly larger. A larger subset size increases the information from the speckle and hence the precision, but at the cost of spatial resolution^27,28^.

The next step is to calculate the correlation coefficient map *γ* of *f* and *g* by using the normalized cross-correlation (NCC)^23,34^

3 $$\gamma \left( {\Delta u,\Delta v} \right) = \mathop {\sum }\limits_{u = - M_f}^{M_f} \mathop {\sum }\limits_{v = - N_f}^{N_f} \frac{{\left[ {f\left( {u,v} \right) - \bar f} \right] \times \left[ {g\left( {u + \Delta u,v + \Delta v} \right) - \bar g} \right]}}{{\Delta f \times \Delta g}}$$

Here, *f* ® (*g* ®) and ∆*f* (∆*g*) are the mean and standard deviation of each subset, respectively. ∆*u* and ∆*v* may assume integer values from −(*M*_*g*_ − *M*_*f*_) to (*M*_*g*_ − *M*_*f*_) and from −(*N*_*g*_ *−* *N*_*f*_) to (*N*_*g*_ *−* *N*_*f*_), respectively. Through this normalization, the correlation coefficient is made independent of the average intensity reaching the camera and of any pixel-to-pixel variations in gain and well capacitance in the camera’s sensor chip.

A sub-pixel registration algorithm allows the generalization of ∆*u* and ∆*v* to real values. It is applied to calculate the real-valued shifts ξ^*u*^ along the *u* direction and ξ^*v*^along the *v* direction that would maximize *γ*(∆*u*,∆*v*). *γ*_max_ is defined as the resulting estimated maximum value. Due to the inherent 2D nature of the speckle pattern, both the speckle displacements ξ^*u*^ and ξ^*v*^ are derived simultaneously. Various algorithms have been developed; the choice among them is a challenge in practical implementation because both the accuracy and computational efficiency are crucial. Of the methods presented by Bing et al.^35^, the correlation coefficient curve-fitting method is chosen because it is the simplest and quickest. Its relatively low accuracy is boosted by the iterative normalized cross-correlation (INCC) procedure, which runs as follows:

- Calculate the correlation coefficient map γ_1_(∆*u*, ∆*v*) of the reference subset f and the deformed subset g from Eq. (3). Define *f*_1_ = *f*.
- Apply the chosen sub-pixel registration algorithm to γ_1_(∆*u*,∆*v*) to calculate ξ^*u*1^ and ξ^*v*1^, the real-valued shifts of *f*_1_ with respect to g that would maximize γ_1_(∆*u*,∆*v*).
- Apply the Fourier shift theorem to generate a new, shifted reference image according to the following rule^36^.

4 $$f_{j + 1}\left( {u,v} \right) = {{{\mathcal{F}}}}^{ - 1}\left\{ {{{{\mathcal{F}}}}\left[ {f_j\left( {u,v} \right)} \right] \cdot \exp \left[ {2\pi i\left( {\frac{{k\xi ^{uj}}}{M} + \frac{{l\xi ^{vj}}}{N}} \right)} \right]} \right\}$$

where ξ^*uj*^and ξ^*vj*^ are the speckle displacements required to bring f_j_ onto *g* as calculated using the correlation coefficient curve-fitting method. *M* and *N* are the image dimensions, and (*k,l*) represent the reciprocal space coordinates corresponding to (*u,v*).

- Repeat with the original subset *f*_1_ replaced by the newly generated shifted subset *f*_j+1_.

In this case, since the displacement between *f*_2_ and *g* is only a few hundredths of a pixel, the tracking errors for the curve-fitting method will be significantly reduced and the procedure will converge quickly. The total speckle displacement is then

5 $$\xi ^{u,v} = \mathop {\sum }\limits_j \xi ^{uj,vj}$$

**Fig. 3:**
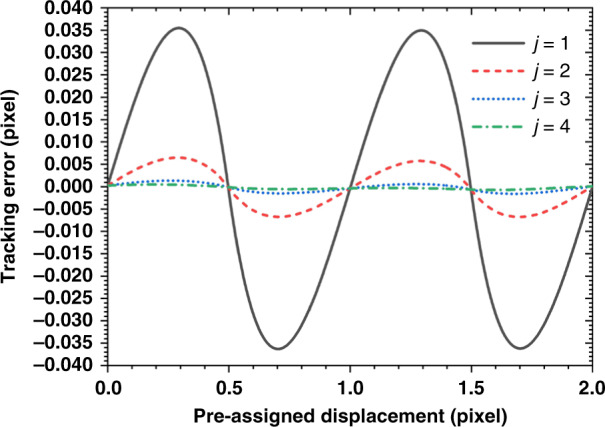
Calculation (tracking) error of the INCC sub-pixel registration algorithms for rigid body translation images over a 0–2 pixel displacement range. The tracking error is reduced significantly by increasing the iteration number j from 1 to 4

The tracking accuracy of the INCC procedure has been verified by shifting a given speckle image by a known preassigned displacement, then applying the procedure to the original image and the shifted image, which take the roles of *f* and *g*. The tracking error is defined as the difference between the preassigned displacement and the shifts ξ^*u,v*^ obtained from INCC^37–39^. Fig. 3 shows that the tracking errors of INCC for *j* = 1,…,4 iterations vary approximately sinusoidally with the preassigned displacement, crossing zero when the preassigned displacement is an integer or half-integer number of pixels. The tracking error for *j* = 1, which is the unmodified correlation coefficient curve-fitting method, can be as high as ±0.035 pixel, which is unacceptably large. However, only a few iterations are required to bring the tracking error down to acceptable levels. As shown in Fig. 3, the maximum tracking errors are reduced to 0.006 pixel and 0.002 pixel after the second and third iterations, respectively. The tracking accuracy can be theoretically pushed below 0.001 pixel after the fourth iteration. However, it should be noted that the speckle noise has not been considered in the above test images. For the real experiment, the tracking accuracy will also be affected by thermal noise, readout noise, and illumination fluctuation^40,41^. A filtering process can be applied to the raw speckle image to reduce the speckle noise before applying the INCC procedure, as shown on a special test mirror that will be examined further in this paper (Supplementary Fig. S3 and S4). Jitter in the placement of the camera pixels can be minimized by the selection of a large area of the camera.

In order to convert the speckle displacement to the deflection angle, the system calibration coefficient *κ* is calculated directly:

6 $$\kappa = P/2d$$

where *d* is the distance traversed by the laser beam from SUT to camera, and *P* is the camera pixel size. As a result, the system sensitivity can be improved with smaller pixel size *P* and larger distance *d*.

In Mode 1, the difference in tangential slope between the region of the SUT covered by *f* and the region of the SUT covered by *g* can be calculated directly from the speckle displacement between *f* and *g*. The calculated speckle displacement from Mode 2 is related to the first derivative of the slope; therefore, the sum of the speckle displacements over all image subsets from *f* to the final *g* yields the difference in tangential slope between the region of the SUT covered by *f* and that covered by the final g. If *f* covers the region around (*x* = 0,*y*) and the final *g* covers the region around (*x,y*), the change *S*^*x*^(*x,y*) in tangential slope encountered in scanning from *f* to the final *g* is given for both modes by

7 $$\left\{ {\begin{array}{*{20}{c}} {S_1^x\left( {x,y} \right) = \kappa \xi _1^v\left( {x,y} \right) + \Delta \phi \left( x \right)} \\ {S_2^x\left( {x,y} \right) = \kappa {\sum} {\xi _2^v} \left( {x,y} \right) + \Delta \phi \left( x \right)} \end{array}} \right.$$

where the subscript 1 and 2 represents Mode 1 and 2, respectively.

For instance, the mirror slope ${S}_{2}^{x}{(x,y)}$ and local radius of curvature *R*(*x,y*) can be approximated as

8 $$S_2^x\left( {x,y} \right) \approx \frac{x}{{R\left( {x,y} \right)}}$$

By taking Eq. (7) into Eq. (8) while ignoring the stage’s pitch error, one can express the first derivative of the slope and the tracking displacement as

9 $$\frac{{\kappa \xi _2^v\left( {x,y} \right)}}{{\Delta x}} = \frac{1}{{R\left( {x,y} \right)}}$$

Here, Δ*x* is the translation step size. The speckle displacement $\xi_{2}^{v}$ between two adjacent images from Mode 2 is thus inversely proportional to the local radius of curvature *R*. If there is a discontinuity in the slope of the SUT, Mode 1 will have a tracking error only at that discontinuity because all images are compared to the same reference and the retrieved displacements for all positions along the mirror are mutually independent. In contrast, because Mode 2 requires integration along the mirror’s length, a discontinuity in slope at one point will affect the slope measurement at other positions too. In principle, both modes can be used for the metrology of plane mirrors or weakly curved mirrors. However, it will be difficult to track the speckle displacement with Mode 1 if the mirror slope varies too widely, as is true if the mirror is strongly curved. Because the speckle displacement is then dramatically larger, Mode 1 will take much more computation time than Mode 2, especially when processing the 2D map. In contrast, Mode 2 measures the differential of the slope, and the speckle displacement between two consecutive images can be precisely tracked.

Existing metrology techniques use a range of optics, such as reflectors, lenses, and pentaprisms, to transport the beam from the source to the mirror surface, and the camera. These optics are inevitably imperfect. Even the very best (λ/100) optics produces sufficient optical path variations to interfere with the most demanding mirror figure error measurements of 1 nm resolution^42^. The use of such optics is therefore kept to a minimum, with only a beam-splitter being used for the present SAM design. In addition, any beam lateral motion in the optical system produces significant slope error for testing a curved surface^42^. Therefore, the SAM instrument is fixed on the carriage and scanned along the translation stage to probe the surface slope, keeping the distance between the surface and camera fixed, avoiding beam lateral motion. While an autocollimator (AC) uses a multislit reticle as a beam marker to detect the deflection of the returned beam from the SUT, a speckle pattern is composed of multiple-beam markers, each carrying unique features. The speckle pattern from the subset window can be tracked with high accuracy by using the proposed INCC procedure. In contrast to a 1D line profile provided by the LTP and the NOM, SAM provides a 2D map of the slope in a single scan by dividing each speckle image into multiple subset windows and performing the pixel-wise analysis perpendicular to the scanning direction. Moreover, unlike the LTP and the NOM, SAM is able through the proposed data analysis procedure (Mode 2) to test strongly curved mirrors by measuring the first derivative of the slope.

## Results

To validate the principle of SAM, an experiment was carried out at the Diamond Optical Metrology Laboratory. The whole system is contained in a thermally isolated enclosure, and the rms value of the temperature variation inside the enclosure is about 0.004 °C over 20 h. (Supplementary Fig. S6). As shown in Fig. 1, the SAM instrument is attached to the air-bearing linear translation stage that is used for the Diamond NOM^12^. Two X-ray mirrors are purposely placed face-up. One high-quality elliptical X-ray mirror polished with the EEM technique is characterized. This mirror belongs to a Kirkpatrick-Baez (KB) pair, a configuration widely used at synchrotrons and free-electron lasers for micro- or nanofocusing. As described in earlier papers^43,44^, a surface profile composed of parabolic sections is added on top of the ellipse, and a uniform top-hat-like power distribution is expected to be generated at the focus. Consequently, the slope profile of the parabolic sections is a series of triangles, which is convenient for demonstrating the functionality, repeatability, and angular sensitivity of SAM. In addition, a cylinder polished with the IBF technique is used to demonstrate the ability of SAM to measure strongly curved mirrors. The stability of the SAM system is measured without moving the SAM optical head, and both horizontal and vertical stability have reached 12 nrad. (Supplementary Fig. S7).

The surface dimensions of the elliptical mirror are 100 mm × 40 mm with 90 mm × 7 mm clear aperture. The ellipse parameters are *p* = 45 m, *q* = 0.4 m, and *θ* = 3 mrad, where *p* is the distance from the light source to the mirror center, *q* is the distance from the mirror center to the focus, and *θ* is the grazing angle. The modulation length of the parabolic sections is 50 mm, and there are two modulations with an amplitude of 50 nm. The exposure time for each speckle image is 1 s, and it takes 37 mins to complete each scan of 486 images over the mirror surface with a translation step size of Δ*x* = 0.2 mm.

**Fig. 4:**
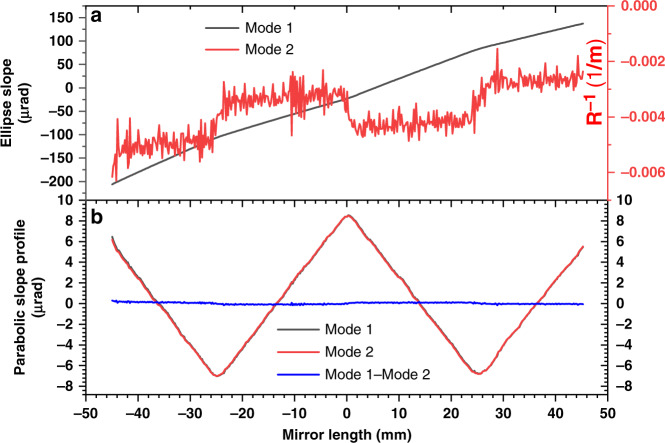
Examples for comparison between 2 data processing modes. **a** The measured tangential slope and reciprocal local radius of curvature with Mode 1 and Mode 2, respectively, of the elliptical mirror with superimposed parabolic arcs. **b** The retrieved slope profile after subtraction of the best-fit ellipse as measured by Mode 1 and Mode 2, and the difference between them

Since the mirror is weakly curved, the speckle displacement over the full mirror can still be tracked with Mode 1. To compare the merits and the limitiations of the two modes, both Mode 1 and Mode 2 are used for the data analysis of this elliptical mirror. Fig. 4 shows the measured results for this elliptical mirror with the SAM. As discussed above, both modes have been used to analyze the data along the central line of the sampled region. The region of interest (ROI) of the selected speckle subset is 1850 pixel (*W*)× 500 pixel (*H*) with an iris aperture D of 4 mm. As shown in Fig. 4a, the mirror slope and the reciprocal local radius of curvature were directly retrieved from the speckle images by using the two modes. The INCC procedure was applied for both modes with three iterations (*j* = 3), and the effectiveness of the INCC procedure has been demonstrated in the Supplementary Fig. S5. Here, the central speckle image is used as the reference to minimize the speckle tracking range for Mode1. The slope changes by more than 343 μrad over the active length of 90 mm, corresponding to an average curvature of under 265 m. This is consistent with the theoretically calculated tangential curvature of 264.3 m at the center. As described in Eq. (7), the slope profile can be calculated by integrating the reciprocal local radius of curvature for Mode 2. The slope profiles retrieved using both the modes were then fitted to an ellipse of fixed parameters *p* = 45 m and *q* = 0.4 m. The value of θ for the best-fit ellipse was 2.98 mrad and 2.97 mrad for Mode 1 and Mode 2, respectively. After the subtraction of the best-fit ellipse, the retrieved slope profile of the elliptical mirror for both modes is shown in Fig. 4b. The slope profile data from Mode 2 agree well with those from Mode 1 over the polished area (90 mm), and the standard deviation of the difference (blue dash-dotted line) of the slope profile data between the two modes is 89 nrad RMS. It should be noted that the computation time for Mode 1 is considerably longer than for Mode 2 since a larger searching range is required to track the greater speckle displacements for Mode 1. For instance, it takes 114 and 23 s for the D data analysis (with *j* = 3) with Mode 1 and Mode 2, respectively. Therefore, Mode 2 is used for analysing the speckle images in the following discussion.

**Fig. 5:**
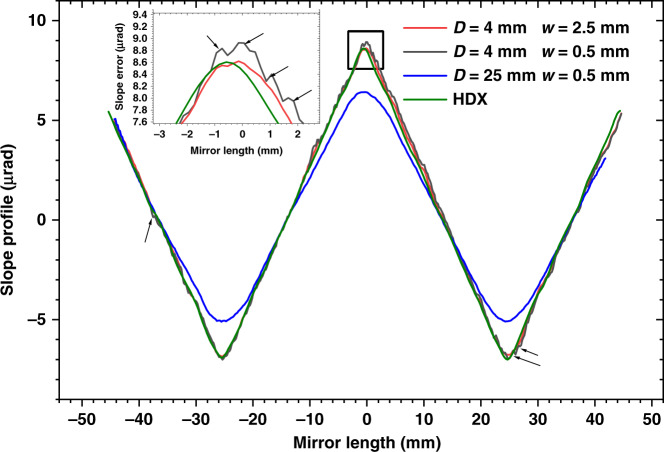
The measured tangential mirror slope profile of the elliptical mirror with SAM and HDX Fizeau interferometer. Each SAM dataset is labelled by its iris aperture *D* and subset window width *w* to demonstrate their effects on the spatial resolution. All SAM measurements use the same subset window length (*l* = 0.5 mm)

Since the object speckle pattern is generated from the diffuser in SAM, it will be essential to reduce the illumination area to improve the spatial resolution. Various iris apertures have been used to demonstrate the effect on the spatial resolution of the SUT. As shown in Fig. 5, the sharp tips of the slope error profile are smeared out when the iris is fully open (*D* = 25 mm) even though a smaller subset window is chosen for data analysis. In this case, the spatial resolution is limited by the large illumination area on the diffuser, and the mirror surface information is convoluted with the speckle patterns. In contrast, the spatial resolution is significantly improved when the aperture is closed to 4 mm. The retrieved slope error data agrees well with the data measured with the HDX Fizeau interferometer (HDX) when a larger subset window (*w* = 2.5 mm) is selected. The HDX data is filtered with spatial resolution of 2–3 mm. The value of *θ* for the best-fit ellipse was 2.97 mrad for the SAM data and 2.99 mrad for the HDX data. The residual slope after subtraction of the best-fit ellipse was 4.346 μrad RMS in the SAM data, which is slightly larger than in the HDX data (4.322 μrad). Although the good agreement is found in most of the regions, there is still some discrepancy between the SAM and HDX data. The rms value of the difference between SAM and HDX data is 146nrad for the case (*D* = 4 mm, *w* = 2.5 mm). The precision of the Fizeau interferometer instrument can be pushed below 50 nrad by using advanced stitching algorithm^45–47^. The precision of the SAM is around 21 nrad for the present setup, (Supplementary Fig. S8) and it can be potential further improved by using dedicated mounting mechanics. It should be noted that the mirror was mounted facing sideways during the measurement with HDX, and the remaining slope error discrepancies between the SAM data and HDX data might come from the nonuniformaity of the slope error along the mirror’s width or the mirror mounting.

When the subset window w is reduced from 2.5 to 0.5 mm, sharp and distinctive features appear, as shown in the inset plot of Fig. 5. Repeated scans show them consistently, proving that they are real features from the mirror and not random noise. This result indicates that the spatial resolution is mainly limited by the subset window size rather than the iris aperture once the iris is closed below a certain width. Further studies of the effects of aperture size and subset window on the spatial resolution will be carried out on mirrors that have features of known size, such as chirped mirrors, which have a sinusoidal pattern of varying spatial frequency at different positions^48^.

Due to the scattering and diffraction of the laser by the diffuser, the speckle beam is divergent from the diffuser up to the mirror and the camera. It is vital to know the resulting beam magnification for mapping the measured reciprocal radius of curvature and its integrated slope to their correct positions onto the mirror surface. The beam magnification from the mirror surface to the camera plane can be approximately expressed with the following beam scaling factor Γ_x,y_:

10 $$\Gamma _{x,y} \approx \frac{L}{{\left( {L - d} \right)}}$$

where *d* and *L* are the beam path lengths from the camera to the mirror surface and to the iris, respectively. However, Eq. (10) is valid only if the illuminated area is assumed planar so that reflection from it does not alter the beam divergence. Moreover, the true scaling factor cannot be accurately calculated with this equation because the measurements of both distance and the reciprocal radius of curvature of the mirror are subject to systematic errors. Here we propose instead to calibrate the beam scaling factor with experimental data taken around a sharp, clearly defined feature.

**Fig. 6:**
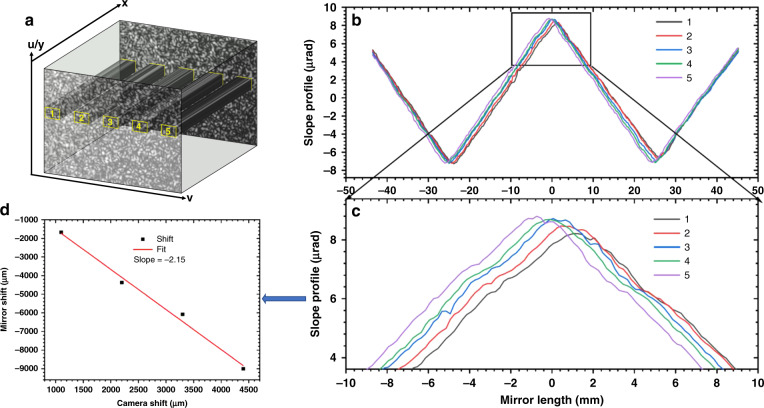
Demonstration of calibration of spatial scaling factor. **a** Schematic representation of the scaling calibration, performed by choosing multiple subset windows at different vertical positions on the camera from a stack of speckle images. **b** The tangential mirror slope profile of the elliptical mirror after subtraction of the best-fit ellipse. **c** The zoomed region of (**b**), showing how the slope error profile shifts with the subset window. **d** The line fitting between the tracked position shift on the mirror and the subset window shift on the camera

As shown in Fig. 6a, the subset window is selected at different positions with constant offset (Δw) along the camera’s height *v*, which corresponds to the scanning direction *x*. As shown in Fig. 6b and the zoomed region in Fig. 6c, the retrieved mirror slope error profile shifts monotonically with different subset window positions (from 1 to 5) once the stacks of speckle images are processed. The mirror coordinates *x* of the retrieved mirror slope error profile is precisely determined by the known scanning step size. In order to calculate the shift between each curve, the INCC approach is also used here, and the displacement between the first position and the other positions can be calculated. Fig. 6d shows the tracked displacement as a function of the subset window offset. As described in Eq. (2), the best-fit slope from Fig. 6d is the magnification ratio between the mirror and camera, and is hence the horizontal beam scaling factor Γ_*x*_. With this approach, the scaling factor can be calibrated based on the measured features from SUT without the need for precise knowledge about the physical distance and the local radius of curvature of the SUT.

**Fig. 7:**
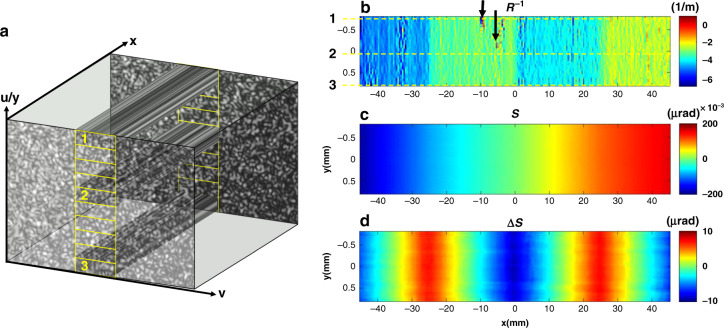
Demonstration of generation of 2D maps of mirror surface. **a** Schematic representation of the generation of 2D maps of the modified elliptical mirror by choosing multiple subset windows along the camera width from a stack of speckle images. The measured 2D map for tangential (**b**) reciprocal local radius of curvature (R^−1^), (**c**) slope (*S*), and (**d**) parabolic modification (∆*S*)

Once the scaling factors along both directions are calibrated, we can divide the speckle image into multiple subset windows and map the retrieved 2D information onto the mirror surface. As illustrated in Fig. 7a, multiple subset windows along the camera width from a stack of speckle images can be processed to generate 2D maps of mirror information. The width *w* of the subset window along *v* will determine the tangential spatial resolution. The length *l* of the subset window along u will be related to the sagittal spatial resolution. The offset *Δl* between consecutive subset windows is set to be the same (as shown in Fig. 7a) or smaller than the length *l* of the subset window. Once the subset window is selected, Mode 2 will be used to track the speckle displacement between two consecutive images with the INCC procedure, and the iterative number is set to three. As described in Eq. (9), a single line profile of the reciprocal local radius of curvature will be generated by a single subset window projected through all the stack of speckle images. Multiple line profiles can be calculated by repeating this process over all subset windows. Fig. 7b displays the 2D map of the retrieved reciprocal local radius of curvature, which is not uniformly distributed along the mirror’s sagittal direction. The three lines marked in Fig. 7b is processed by selected the corresponding region in Fig. 7a. As marked with arrows in Fig. 7b, some structures appear in the reciprocal local radius of curvature, which will be directly linked to the irregular structures of the defocused beam when the mirror is illuminated with X-rays^49^. Therefore, it will be useful to have such 2D maps of the reciprocal local radius of curvature in order to predict the beam performance. The mirror slope can be calculated by integrating the reciprocal local radius of curvature as in Eq. (7). Fig. 7c displays the resulting 2D mirror slope map. For each line along the mirror’s tangential direction, the slope of the best-fit ellipse is calculated and subtracted, and the residual slope error map is shown in Fig. 7d. Evidently, the 2D map provides much richer information than the 1D profile.

To demonstrate the potential application of the proposed technique to strongly curved mirrors, a cylindrical mirror with sagittal radius of curvature 445 mm is measured with SAM. The surface dimension of the cylindrical mirror is 100 mm × 25 mm with 90 mm × 10 mm clear aperture. The measurement is carried out along the mirror sagittal direction, which here is *x*. As explained earlier, Mode 1 no longer works since the speckle displacement is too big to be tracked. Therefore, only Mode 2 is used to track the speckle displacement between consecutive images. As described in Eq. (9), for a cylindrical mirror in the present case, *R* = 445 mm, *κ* = 2.14 μrad/pixel, and the speckle displacment $\xi_{2}^{v}$ is about 1050 pixels between consecutive images if the step size is Δ*x* = 1 mm. Therefore, to reduce the speckle displacement $\xi_{2}^{v}$ for the strongly curved mirror, the step size should be made as small as possible. In this study, the step size is 0.02 mm and 501 images are collected over the 10 mm long clear aperture. Once the speckle displacement is derived with the INCC procedure, the slope and height profiles are then reconstructed by integration.

**Fig. 8:**
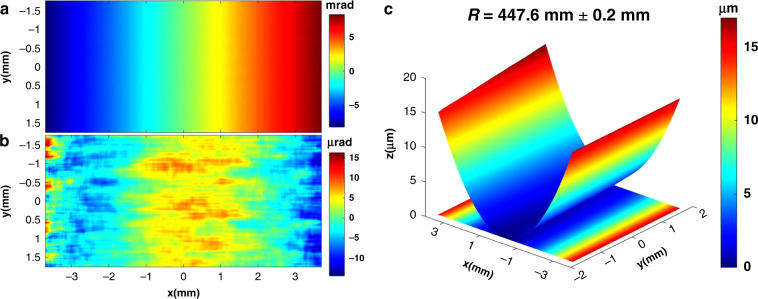
Measured 2D surface profiles of a strongly curved cylinder mirror. **a** Mirror sagittal slope (**b**) mirror sagittal slope error, and (**c**) height profile images of the strongly curved cylinder mirror. The measured mirror slope range is above 16.5 mrad over 7.4 mm, and the measured slope error and the measured radius of curvature of mirror are 4.2 urad and 447.6 ± 0.2 mm, respectively. Note the change of definition: *x* is here the sagittal coordinate and *y* the tangential coordinate of the mirror

Fig. 8a shows the measured mirror sagittal slope, which covers a range of 16.5 mrad over the analysed area. SUTs that vary so widely in slope could not easily be measured on a NOM or an LTP without being tilted between scans or compromising the LTP’s performance. The cylindrical fitting is applied for each pixel along the mirror tangential direction (y). As shown in Fig. 8b, there is clearly a variation of the mirror sagittal slope error along the tangential direction. The mirror slope error is 4.2 μrad, which is smaller than the specified 5μrad. In addition, the fitted radius of curvature of the mirror is 447.6 mm ± 0.2 mm. It indicates that this mirror meets its specification (445 mm ± 5 mm). The constructed mirror height profile is shown in Fig. 8c. The height difference between the mirror’s center and its edge is over 15 μm. Such a steep mirror profile would pose significant challenges for most other metrology techniques.

## Discussion

In summary, we have developed a novel metrology instrument—SAM, which can be easily installed on an existing ex-situ metrology gantry. The SAM instrument is a versatile, compact, low-cost, and ready-to-use metrology instrument. It can generate 2D surface profiles, which provide much rich information about the surface profile of X-ray mirrors. In addition to the larger scanning angle range and excellent repeatability, high accuracy is achieved thanks to the INCC algorithm. Furthermore, with smaller subset window sizes and step sizes, better spatial resolution below 0.5 mm is achievable. Such flexibility will enable SAM-based instruments to perform metrology on mirror surfaces within the most desirable mid-spatial frequency range, which is crucial for simulating the X-ray beam performance^50^.

The SAM instrument can also be used to measure toroidal, ellipsoidal, and paraboloidal mirrors by performing 2D raster scans of SAM across the entire mirror surface. In this case, it will be essential to either rotate the mirror by 90° or scan the mirror along the sagittal direction with an additional autocollimator in order to retrieve the sagittal slope error map. Moreover, the SAM instrument can measure mirrors that are facing up, down, or sideways, as they would be installed on the beamline. Finally, the SAM instrument is not confined to synchrotron X-ray mirrors but can also be applied to freeform optics and to high-quality mirrors in other fields, such as extreme ultra-violet lithography and laser ignition.

After the successful demonstration of this novel instrument of metrology for X-ray mirrors, we plan to improve the device further by enhancing its stability, robustness, data acquisition, and processing. For instance, the present measurement speed is limited due to the significant time taken in using the linear motor in step-scan mode. The measurement speed can be significantly enhanced by applying the advanced fly scan technique^51^. This novel technique of speckle angular measurement can then expand the capabilities of current metrology instruments to new X-ray optics that now cannot be made because they cannot be measured. For the next generation of X-ray mirrors, which will be required to keep up with new X-ray sources and the ever-increasing demand for greater coherence and tighter focusing, SAM will be a timely source of aid.

## Materials and methods

**Fig. 9:**
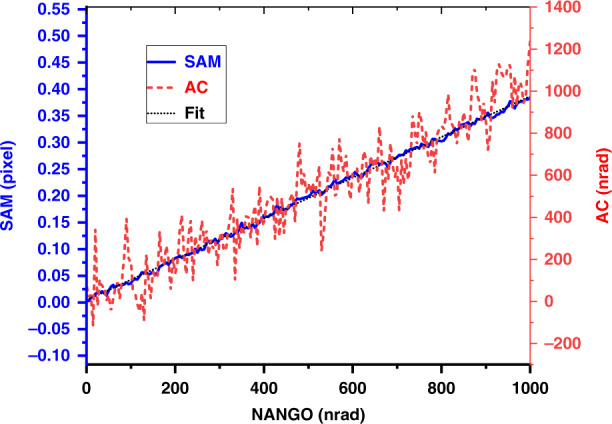
The speckle displacement for SAM and the measured angle change for AC as function of the NANGO angle over 1000nrad. The slope of the best-fit line to the SAM data is 3.82E-4 pixel/nrad

The Diamond nano-angle generator (NANGO)^52^ is used to calibrate the linearity of the SAM system. The NANGO angle is varied with a constant step size of 10nrad, and the speckle images were recorded by SAM. In Fig. 9, the retrieved speckle displacement (in Mode 1) is plotted as a function of the NANGO deflection angle. The best-fit slope is 3.82E-4 pixel/nrad. Here, the calibration coefficient *κ* is the reciprocal of the best-fit slope, and the actual detected angle from SAM is calculated by multiplying the speckle displacement by the calibration coefficient *κ* (2618 nrad/pixel or 2.618 μrad/pixel). As a comparison, the AC is measuring the other side of the NANGO cube mirror simultaneously. Fig. 9 shows the measured angle from the AC. It is clearly seen that the AC data are much noisier than the SAM data. While the standard deviation for the AC is 104nrad, the one for the scaled SAM data is only 10.2 nrad, which is a factor of 10 better. Note that both the AC data and the SAM data were simultaneously measured in the same single scan. It should be noted that we can’t conclude that 10 nard angular accuracy can be achieved by SAM since the generated angular error from NANGO has not been considered. The precision for both the SAM and AC can be further improved by averaging multiple repeated datasets to remove random noise.

**Fig. 10:**
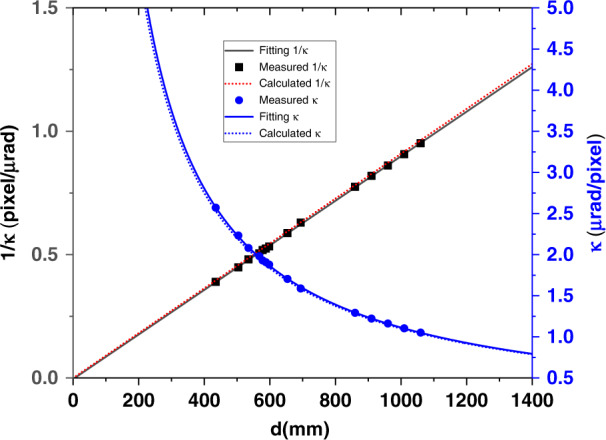
Calculated and measured system calibration coefficient. The calibration coefficient *κ* and its reciprocal as the function of the distance between the mirror surface and the camera

As described in Eq. (6), the system calibration coefficient κ is calculated from the mirror-to-camera distance *d* and the camera pixel size *P*. To verify this equation and fully calibrate the SAM system, NANGO is fixed on a motorized platform, and the distance *d* between the SAM and NANGO can be precisely changed with a linear motor. The above fitting procedure (in Fig. 9) is repeated at a few distances *d*. As expected, the fitting slope 1/*κ* (black dot curve) is linearly proportional to the distance d in Fig. 10. By linearly fitting the 1/*κ* with the distance *d*, we extract the measured distance error Δ*d* = 4.2 mm from the fitting intercept. This error is due to the deviation from the exact camera sensor position. The system calibration coefficient *κ* is plotted as a function of the measured distance *d*, and the calibration coefficient *κ* is used for SAM metrology. For instance, the distance d between the test mirror surface and SAM camera is 514 mm, and the measured calibration coefficient *κ* = 2.140. If the measured distance is 513 mm, the corresponding calibration coefficient *κ* = 2.144. In other words, the calibration coefficient error is then 0.2% if the measured distance error is within 1 mm at this working distance.

In general, four predominant parameters, namely, the tracking accuracy (∆*ϵ*), camera pixel size (*P*), the distance of the beam path from the test surface to the camera (*d*), and the system noise (*Δn*) determine the angular sensitivity *∆σ* of the SAM instrument for mirror metrology. This can be calculated by

11 $$\Delta \sigma = {{\sqrt {\left( {\Delta \smallint P} \right)^2 + \left( {\Delta n} \right)^2} }}/{{2d}}$$

**Fig. 11:**
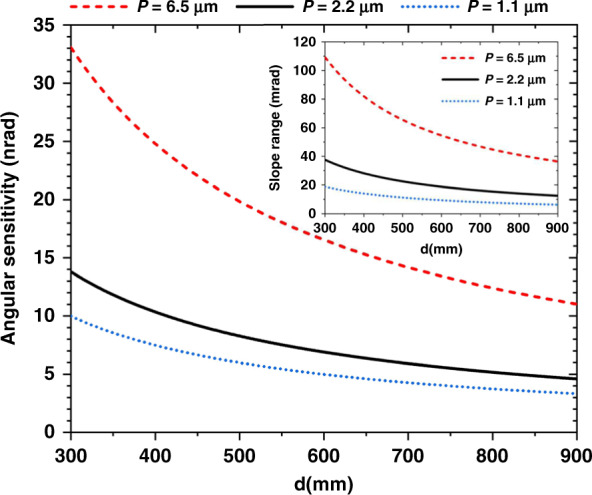
Calculated angular sensitivity and slope range of SAM. The angular sensitivity and the corresponding slope measurement range (inset figure) of SAM as a function of the distance between the mirror surface and camera with different pixel size *P*

The system noise (*Δn*) includes both the instability of the SAM system and the relative vibration between the SAM head and the test mirror. As shown in Fig. 11, better angular sensitivity can be achieved with smaller pixel size and larger distance. The tracking accuracy of the INCC method, which is set by the thermal noise and the readout noise, is estimated to be 0.003 pixel. The system noise, which arises from internal and external vibrations, is estimated at 0.01 μm. All the above calculation is based on the achieved angular sensitivity of about 10 nrad at 514 mm. Here, the angular sensitivity is mainly limited by the system noise rather than the tracking accuracy. When the pixel size is reduced from 2.2 to 1.1 μm, the angular sensitivity fails to improve proportionally. However, the angular sensitivity can be improved by averaging a series of repeated speckle imaging scans to smooth out random noise^30^.

In addition to the higher angular precision, a larger measurement slope range *Ω* is also desirable for measuring strongly curved mirrors. For the SAM system, this range is equal to the beam acceptance angle for the camera: as long as the reflected beam can be detected by the camera, the speckle displacement can be tracked with the proposed Mode 2. *Ω* is determined by the camera chip size *CS*_cam_ and the distance d between the camera and test mirror surface:

12 $${{\varOmega }} = \frac{{2CS_{cam}}}{d}$$

In the inset graph of Fig. 11, the camera has 2560 pixels along the scanning direction. The camera chip size is this number of pixels times the pixel size. As expected, the larger pixel size can provide a larger slope range. For instance, the slope range is about 22 mrad for the present *P* = 2.2 μm at 514 mm, but it can be increased up to three times by using a pixel size *P* = 6.5 μm with reduced angular sensitivity. Moreover, the slope range can be further increased by reducing the distance from the SAM head to the mirror, which can be adjusted for the given experimental setup.

## Supplementary information

Supplementary Information for Nano-precision metrology of X-ray mirrors with laser speckle angular measurement
