# Supplementary material for: Nano-precision metrology of X-ray mirrors with laser speckle angular measurement
Source: Light Sci Appl. 2021 Sep 22;10:195. doi: 10.1038/s41377-021-00632-4 (PMC8458457; doi:10.1038/s41377-021-00632-4)
Supplement: Supplementary file 1 — Supplementary Information for Nano-precision metrology of X-ray mirrors with laser speckle angular measurement [file 41377_2021_632_MOESM1_ESM.docx]

**Supplementary information for**

**Nano-precision metrology of X-ray mirrors with laser speckle angular measurement**

Hongchang Wang^1^*, Simone Moriconi^1,2^ and Kawal Sawhney^1^

^1^Diamond Light Source Ltd, Harwell Science and Innovation Campus, Didcot, OX11 0DE, UK

^2^Department of Engineering Science, University of Oxford, Parks Road, Oxford, OX1 3PJ, UK

1. *Correspondence: Hongchang Wang (email: hongchang.wang@diamond.ac.uk)

# **Section-1: Experimental set-up**


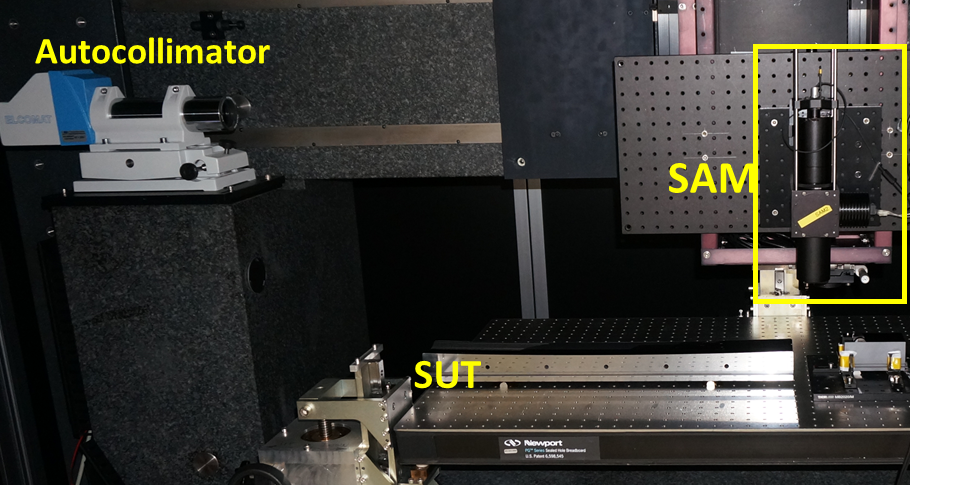


Figure S1. The photo of the SAM mounted on the Diamond-NOM gantry for metrology of the X-ray mirror mounted on the granite table. The X-ray mirror is the surface under test (SUT). The NOM’s carriage slide is at top. The autocollimator for measuring the pitch error of the carriage slide is at upper left. The SAM head is at right.

# **Section-2: Mean intensity gradient (MIG)**


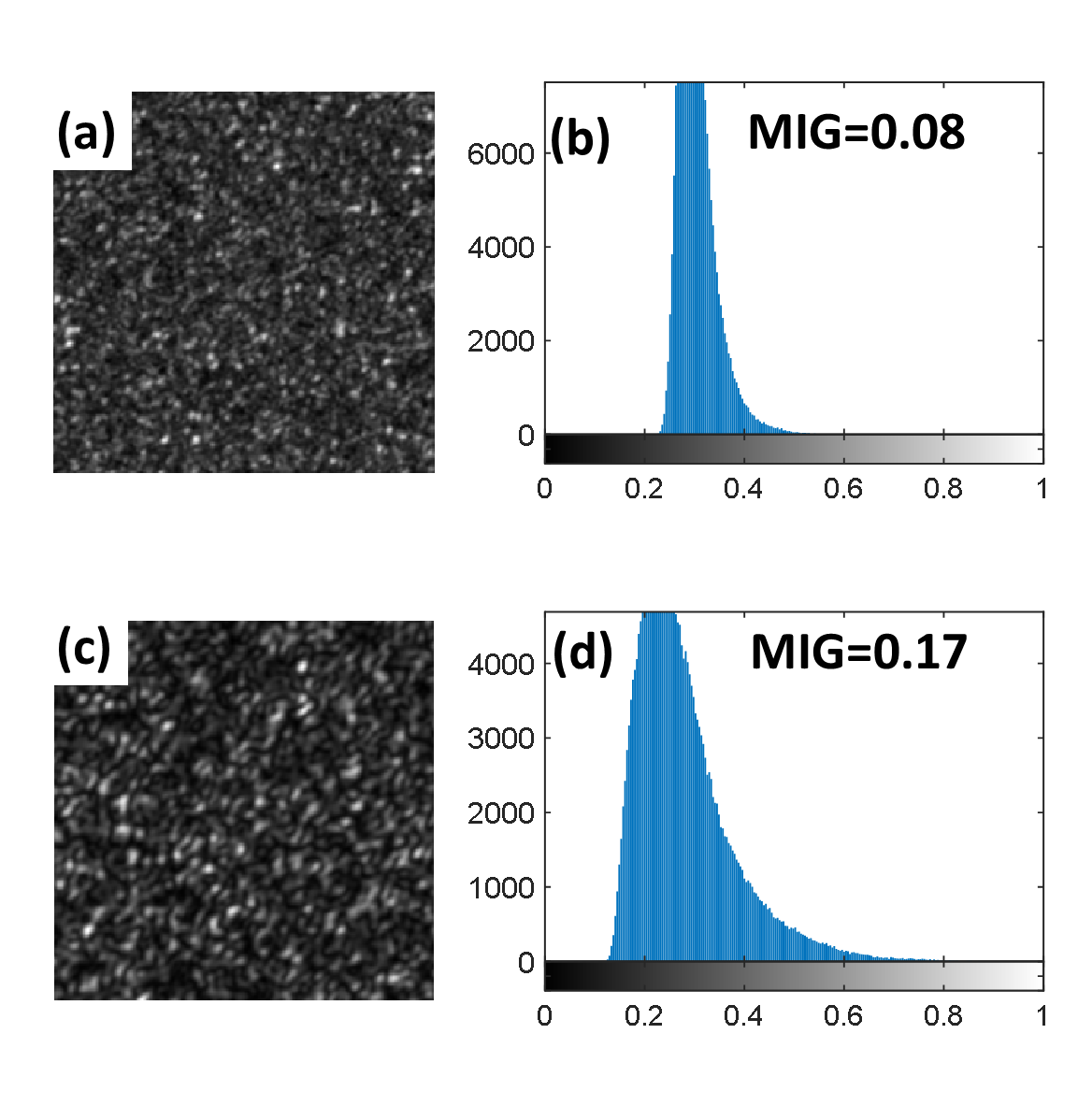


Figure S2. The speckle patterns and their corresponding histograms with (a and b) ground glass diffuser with 600 grit size (c and d) invisible tape

The Mean Intensity Gradient (MIG) is used to assess the overall quality of different speckle patterns.^1^ The MIG is defined as follows:

$$\delta_{f}=\sum_{i=1}^{W} \sum_{j=1}^{H} \sqrt{{f_{x}\left( x_{ij} \right)}^{2}{{+f}_{y}\left( x_{ij} \right)}^{2}}$$

where W and H (in unit of pixels) are image width and height, and $f_{x}\left( x_{ij} \right)$ and $f_{y}\left( x_{ij} \right)$ are the x- and y-directional intensity derivatives at pixel $\left( x_{ij} \right).$

As shown in Fig. S2, the MIG of the speckle patterns generated by invisible tape is nearly twice that of the speckle patterns generated by the ground glass diffuser. In addition, the visibility of the speckle pattern, defined as the ratio between the standard deviation and the mean, is only 0.12 for the ground glass diffuser but 0.35 for the invisible tape. The higher visibility is very beneficial for achieving higher tracking accuracy.

# **Section-3: Specification for the parabolic section**

Figure S3. Specified surface slope and height profiles of the parabolic section superimposed on the test elliptical mirror (after ellipse subtraction).

As shown in Fig. S3, an additional height profile is added along the tangential direction of the surface of an elliptically figured mirror. It can shape the X-ray beam to a top-hat profile with 10 μm width in the focal plane.^2,3^ Each half wave is a separate parabolic arc. The height profile function and its first derivative are continuous at the junction between parabolic arcs. The modulation length of the parabolic sections is 50 mm, and there are 2 modulations along the full mirror length with a modulation amplitude of 50 nm.

# **Section-4: Gaussian filter to remove noise**

Figure S4. (a) raw speckle image (No filter) and the processed speckle image with Gaussian Filter (b) Sigma=5 and (c) Sigma=10. (d) the retrieved slope profile of parabolic arcs in Fig. S3 with the above three types of speckle images.

The speckle image contrast is affected by the thermal noise, readout noise and illumination fluctuation.^4,5^ A filter must therefore be applied to the raw speckle image to smooth out the noise (Fig. S4(a)-(c)). Fig. S4(d) shows the retrieved slope profile of the parabolic arcs superimposed on the test elliptical mirror. If unfiltered speckle is used, the retrieved profile is a very poor match to the true profile, but the application of Gaussian filters to the speckle produces much better agreement.

# **Section-5: Iterative normalized cross-correlation (INCC)**

Figure S5. (a) the slope profile of the parabolic arcs on the test elliptical mirror as measured in Mode 2 using the INCC procedure with 1, 2 or 3 iterations (INCC1, INCC2 and INCC3, respectively). (b) the change of the measured slope profile from the first to the second iteration (INCC2-INCC1) and from the second to the third iteration (INCC3-INCC2).

The proposed INCC procedure is applied with 1, 2 or 3 iterations to demonstrate the importance of better tracking accuracy for precision metrology. As shown in Fig. S5(a), the retrieved slope profiles of the parabolic arcs on the test elliptical mirror appear independent of the number of iterations. However, in Fig. S5(b), when the slope profile for the first iteration is subtracted from the slope profile for the second iteration, one finds that the slope profiles differ by as much as −0.08 µrad to +0.14 µrad. This demonstrates the importance of applying multiple iterations, but the rapid convergence of the INCC procedure is shown by the negligible difference between the second and third iterations. Once convergence is achieved, the tracking accuracy is limited not by the tracking error but by speckle noise, which can be smoothed out with a Gaussian filter as shown in Fig. S4.

# **Section-6: Temperature stability**


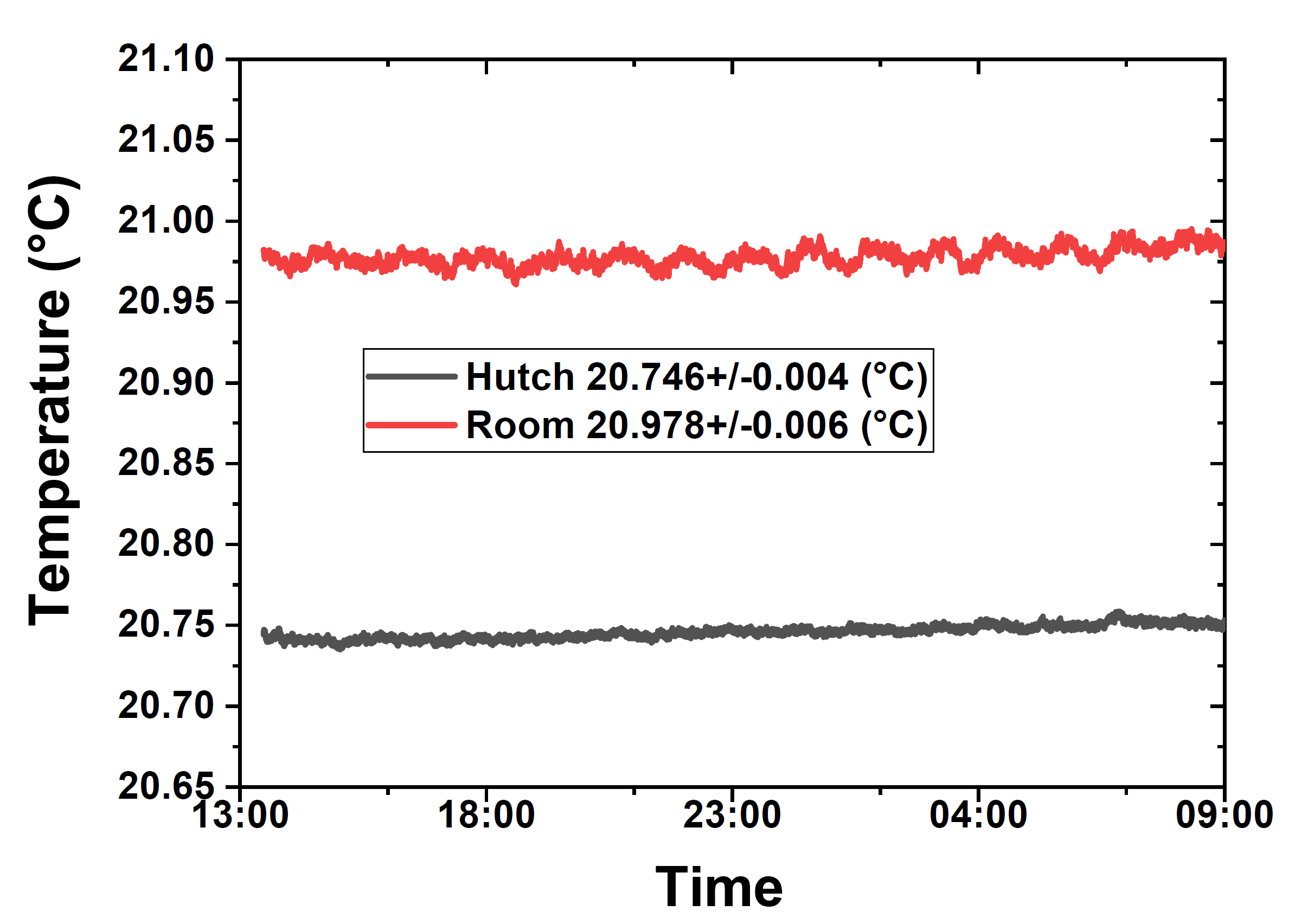


Figure S6. The room and hutch temperature of the metrology lab over 20 hours.

In order to provide stable measurement conditions, the whole system is contained in a thermally isolated enclosure. The temperature in the enclosure is not actively stabilized, and it relies simply on the huge heat capacity of the granite bench and the very small power dissipation inside it. Fig. S6 shows that the standard deviation of the temperature variation in the optics metrology room is 0.006 °C, while the one in the enclosure is 0.004 °C over 20 hours. The stable temperature also minimizes air currents, which affect the stability of the speckle pattern.

# **Section-7: SAM system stability**


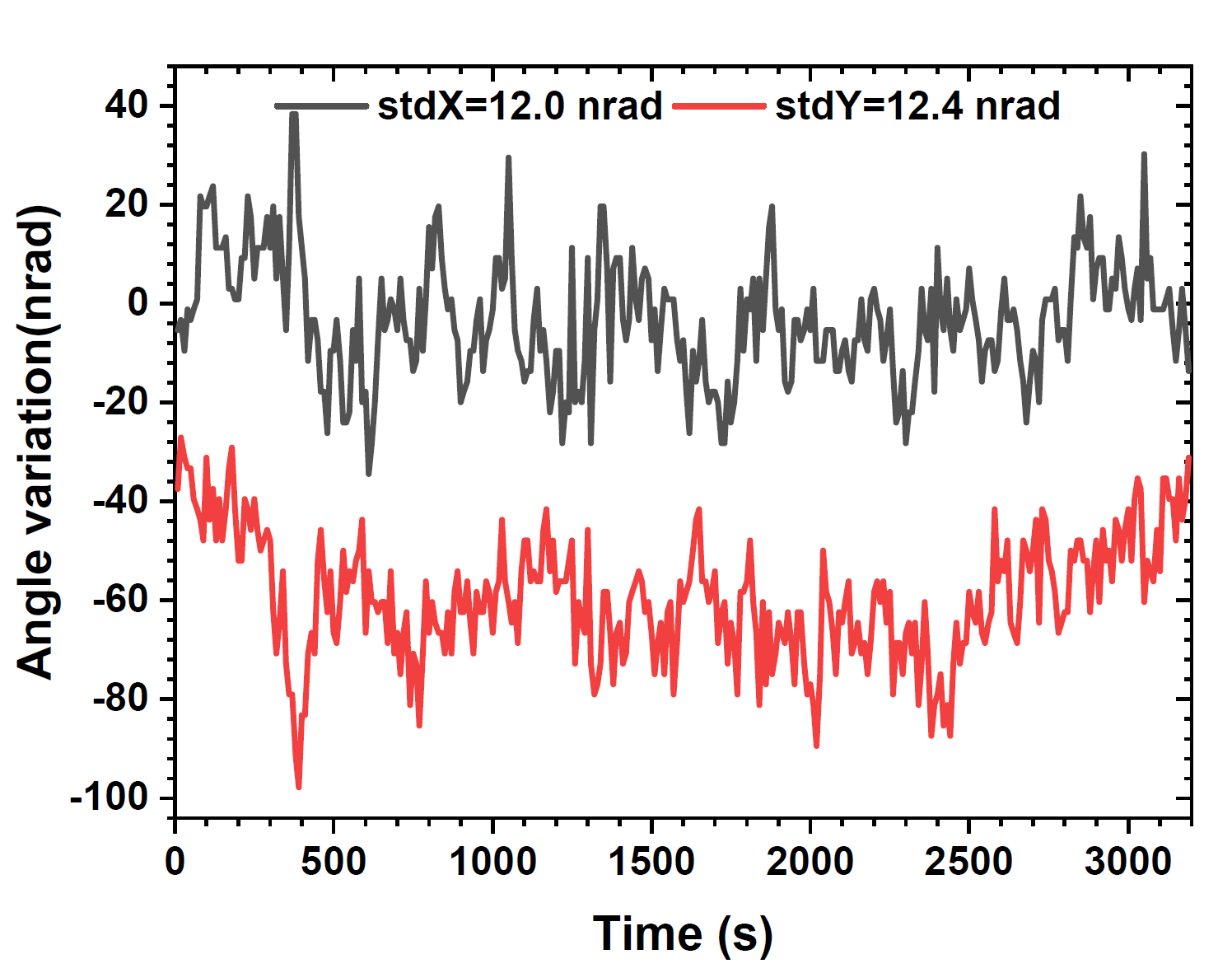


Figure S7. The stability measurement for SAM over 3000 s

To check the noise level of the SAM system, the stability scan is carried out without moving the SAM optical head. As shown in Fig. S7 both horizontal and vertical stability have reached the 12nrad level. If we assume the speckle tracking accuracy is 0.003 pixel, which is equivalent to 6 nrad in this configuration, the precision of the present SAM system is limited by the vibrational stability rather than the tracking accuracy.

# **Section-8: Repeatability measurement**

Figure S8. The other lane of the elliptical mirror, which does not have the parabolic section, is measured 10 times. The line profiles of the tangential mirror slope error are shifted by 100nrad for clarity.

To check the repeatability of the proposed SAM method, the other lane of the elliptical mirror, which does not have the parabolic section, is measured 10 times. The slope for each scan is calculated using Mode 2. Figure S8 shows the difference between the slope of each of the individual scans and the average slope of the 10 scans. The calculated rms value for each of these 10 difference curves is listed on the right side of the graph. The averaged rms value is 21 nrad over the 10 scans. This demonstrates the excellent environmental stability and repeatability of the SAM technique.

**Reference:**

1 Pan, B., Lu, Z. X. & Xie, H. M. Mean intensity gradient: an effective global parameter for quality assessment of the speckle patterns used in digital image correlation. *Optics and Lasers in Engineering* **48**, 469-477 (2010).

2 Laundy, D. *et al.* Surface profiling of X-ray mirrors for shaping focused beams. *Opt. Express* **23**, 1576-1584 (2015).

3 Laundy, D. *et al.* Development of a multi-lane X-ray mirror providing variable beam sizes. *Rev. Sci. Instrum.* **87**, 051802 (2016).

4 Liu, S. *et al.* sCMOS noise-correction algorithm for microscopy images. *Nat. Methods* **14**, 760-761 (2017).

5 Mandracchia, B. *et al.* Fast and accurate sCMOS noise correction for fluorescence microscopy. *Nature Communications* **11**, 94 (2020).
